# Modelling Cell Origami via a Tensegrity Model of the Cytoskeleton in Adherent Cells

**DOI:** 10.1155/2019/8541303

**Published:** 2019-08-14

**Authors:** Lili Wang, Weiyi Chen

**Affiliations:** ^1^Shanxi Key Laboratory of Material Strength & Structural Impact, College of Biomedical Engineering, Taiyuan University of Technology, Taiyuan 030024, China; ^2^National Demonstration Center for Experimental Mechanics Education, Taiyuan University of Technology, Taiyuan 030024, China

## Abstract

Cell origami has been widely used in the field of three-dimensional (3D) cell-populated microstructures due to their multiple advantages, including high biocompatibility, the lack of special requirements for substrate materials, and the lack of damage to cells. A 3D finite element method (FEM) model of an adherent cell based on the tensegrity structure is constructed to describe cell origami by using the principle of the origami folding technique and cell traction forces. Adherent cell models contain a cytoskeleton (CSK), which is primarily composed of microtubules (MTs), microfilaments (MFs), intermediate filaments (IFs), and a nucleoskeleton (NSK), which is mainly made up of the nuclear lamina and chromatin. The microplate is assumed to be an isotropic linear-elastic solid material with a flexible joint that is connected to the cell tensegrity structure model by spring elements representing focal adhesion complexes (FACs). To investigate the effects of the degree of complexity of the tensegrity structure and NSK on the folding angle of the microplate, four models are established in the study. The results demonstrate that the inclusion of the NSK can increase the folding angle of the microplate, indicating that the cell is closer to its physiological environment, while increased complexity can reduce the folding angle of the microplate since the folding angle is depended on the cell types. The proposed adherent cell FEM models are validated by comparisons with reported results. These findings can provide theoretical guidance for the application of biotechnology and the analysis of 3D structures of cells and have profound implications for the self-assembly of cell-based microscale medical devices.

## 1. Introduction

Cell origami is defined as a technique that harnesses the traction force of living cells as a biological driving force to fold a variety of three-dimensional (3D) cell-populated microstructures [1]. In the field of microfabrication, the origami folding technique has received increasing attention due to its multiple advantages, including simplicity, high biocompatibility, the lack of special requirements for substrate materials, and the lack of damage to cells. For example, Davis et al. [2] and Azam et al. [3] used surface tension to create microsized containers. Sirrine et al. [4] and Song et al. [5] used the same technique to produce artificial tissue scaffolds. In addition, Kaori et al. [1] experimentally determined that cells applied the principle of the origami folding technique and cell traction forces to fold many microstructures from two-dimensional (2D) to 3D. Recently, He et al. described an origami-inspired self-folding method to form 3D microstructures of cocultured cells and indicated that the origami-based cell self-folding technique is useful in regenerative medicine and the preclinical stage of drug development [6]. However, none of these studies have investigated cell origami by using the finite element method (FEM).

Cell traction forces, as the contractile forces pointing to the centre of the cell body, are generated by the cytoskeleton (CSK) [7]. The CSK is a complex biopolymer network composed of microtubules (MTs), microfilaments (MFs), and intermediate filaments (IFs) [8]. The CSK is the major mechanical component of cells and plays a key role in mechanotransduction and extracellular force transmission from/to attaching a substrate through focal adhesion complexes (FACs) [9]. The forces in the CSK are related to the biological functions of cells, such as differentiation, growth, metastasis, and apoptosis [10–13]. The nucleus is regarded as an integral structure functionally enabled by nuclear tensegrity, with struts representing the nuclear lamina and cables representing chromatin [12], featuring a large volume occupancy and including genetic information [14]. For example, Bursa et al. simulated the nucleoskeleton (NSK) as a tensegrity structure to study the CSK to transfer the external mechanical load of the cell to NSK, thereby initiating the biochemical response of the cell [15]. In addition, the important role of the NSK in cellular differentiation and development has been demonstrated [16]. Since some researchers [9, 10, 17–28] have used both the spherical and flattened tensegrity structure models' approach combined with computational and mathematical models to investigate the responses of cells to the substrate based on the assumption that individual cells can react by contraction and that the forces produced by cells can act on the extracellular matrix (ECM) by FACs. There are many cell models and models of cell-substrate interactions; however, FEM simulation of cell origami has never been performed. Therefore, it is necessary to establish a simple 3D FEM model of adherent cells composed of the CSK and NSK based on the tensegrity structures to simulate the cell origami.

In this study, a 3D FEM model of an adherent cell made up of the CSK and NSK is established and connected with a microplate to depict the cell origami. The CSK and NSK are represented by tensegrity structures of different levels of complexity, in which the CSK is composed of MTs, MFs, and IFs and the NSK consists of the nuclear lamina and chromatin. The cell model adheres to the microplate through the spring elements representing FACs [29, 30]. The effects of the level of complexity on the folding degree of the microplate are investigated by changing the degree of complexity of the cell tensegrity structure. Furthermore, the role of the NSK is studied by using a 12-node and a 24-node sphere-like tensegrity structure in comparison with the models without the NSK. The validity of the proposed models is validated by comparisons with the reported findings, demonstrating that the models can provide an attempt to measure the cell traction force in a 3D physiological environment and a new way of promoting a deeper understanding of cell origami.

## 2. Materials and Methods

### 2.1. Tensegrity Model

The process of cell origami is modelled by using the adherent cell to fold the microplate. Geometries of the models are created using UG NX 10.0 (Unigraphics NX 10.0) and then imported into the commercial finite element package ABAQUS (standard version 6.13, SIMULIA company, Germany) for simulations and analysis. Since some studies have shown that IFs, as one of the major structure components of the CSK, play important roles in biological functions such as cell contractility, migration, stiffness, and stiffening [31]; play key mechanical roles in providing structural stability of the cell; and can increase the cellular rigidity at high strains (>20% strain) [24, 32]. Moreover, in view of the fact that cell origami is a large deformation process and IFs can provide resilience against mechanical forces and ensure cellular integrity [33], it is necessary to consider the role of IFs. Computational models of adherent cells composed of the CSK which is made up of MTs, MFs, and IFs and the NSK which mainly consists of the nuclear lamina and chromatin have been developed based on the tensegrity structure. Since IFs vary from cell type to cell type [33] and some studies have shown that IFs form a dense filament network spanning from the nucleus to the cell membrane [34], IFs are modelled as radial cables from the centre of the tensegrity structure to the outer nodes in the models.

**Figure 1:**
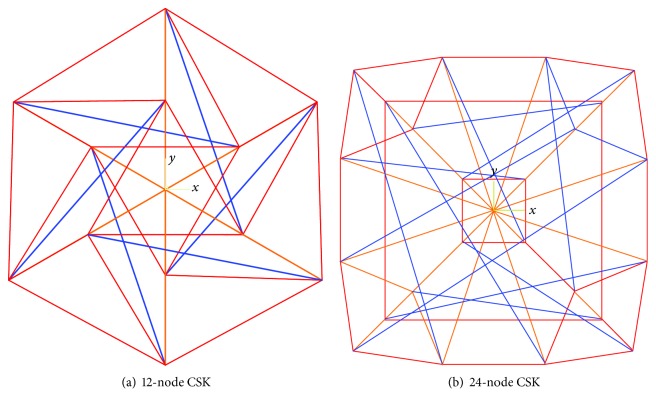
Schematic diagram of the CSK with two different levels of complexity: (a) the 12-node CSK is composed of 6 struts (blue), 24 cables (red), and 12 cables (yellow); (b) the 24-node CSK is made up of 12 struts (blue), 36 cables (red), and 24 cables (yellow).

Although the sphere-like tensegrity structure model derived from the polyhedron (cuboctahedron or octahedron) [9] is symmetrical, the flat tensegrity structure derived from the truncated polyhedron is not completely symmetric. Two asymmetrical tensegrity structures derived from the truncated polyhedron (flat cuboctahedron or octahedron), 12-node tensegrity and 24-node tensegrity, are established to represent the different levels of complexity of the adherent cell models. The 12-node tensegrity structure is composed of 6 struts representing MTs and 36 cables, 24 of which represent MFs and 12 of which represent IFs, as shown in Figure 1(a). The number of cables in the 24-node tensegrity structure is 60, and the number of struts is twice that of the 12-node tensegrity structure. Among the 60 cables, 36 cables represent MFs, whereas the other 24 cables represent IFs, as demonstrated in Figure 1(b). In both tensegrity structures, the cables (red elements) representing MFs are connected by the nodes at both ends of the struts (blue elements), while the cables (yellow elements) representing IFs are connected by the nodes at one end of the struts and the particle at the centre of the structures which is simplified by the nucleus.

**Figure 2:**
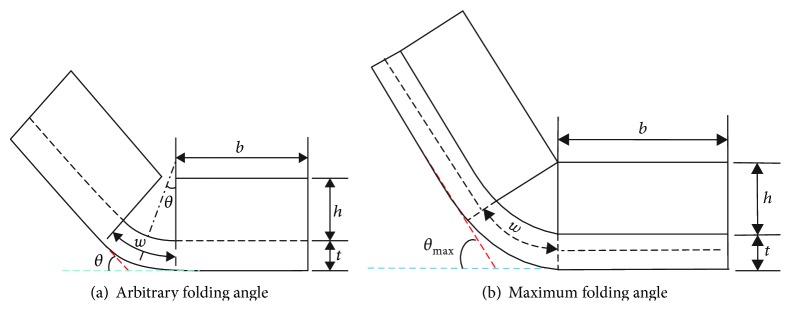
Schematic diagram of the folding angle of the microplate: the horizontal blue dashed line represents the initial position of the microplate; the oblique red dashed line represents the position of the folded microplate.

The microplate on which the cell is adhered is treated as an isotropic linear-elastic solid material, and its dimensions are adapted to the cell model. The microplate has a length (*b*) and a width (*b*) of 30 *μ*m and a height (*h*) of 2.7 *μ*m; furthermore, the dimensions of the joint are 6 *μ*m in width (*w*), 30 *μ*m in length (*b*), and 0.*3 μ*m in thickness (*t*), which shows that the flexible joint is 3~8 *μ*m in width and 70~390 nm in thickness [1]. The spring elements are selected to link the cell model with the microplate. The folding angle (*θ*) is defined as the angle between the folded microplate and its initial position, which is an important parameter for producing desired 3D cell-populated microstructures, as shown in Figure 2.

From a geometric point of view, the folding angle can be expressed by the thickness (*t*) and the width (*w*) of the joint and the thickness of the microplate (*h*) as follows [1]:

(1) $$\begin{array}{c} \theta_{\max}=\frac{w}{h+\left(t/2\right)}, \end{array}$$

When *w* is 6 *μ*m, *h* is 2.7 *μ*m, and *t* is 0.3 *μ*m, the maximum folding angle is 120° according to equation (1). The following folding angle must be less than the aforementioned value (<120°) since the cell traction force of the measurement is inaccurate when the microplate contacts.

**Figure 3:**
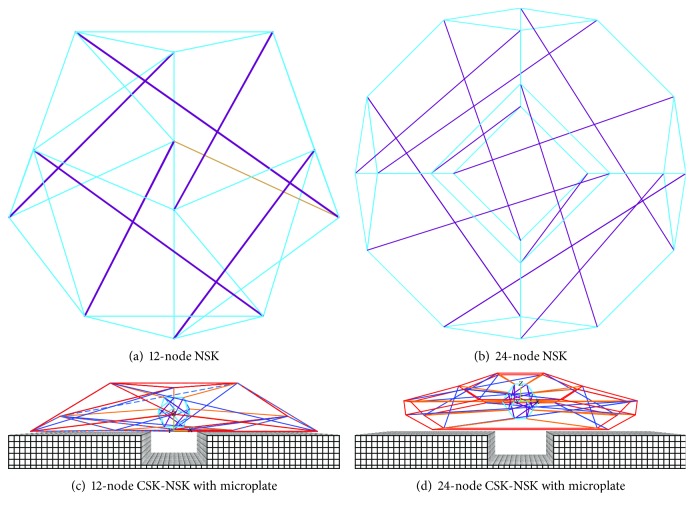
Schematic diagram of tensegrity structures with two different levels of complexity: (a) the 12-node NSK is composed of 6 struts (purple) and 24 cables (cyan); (b) the 24-node NSK is made up of 12 struts (purple) and 36 cables (cyan); (c) the 12-node CSK-NSK with a microplate; (d) the 24-node CSK-NSK with a microplate.

Furthermore, in order to simulate the influence of the NSK on the folding angle, the 12-node (Figure 3(a)) and 24-node (Figure 3(b)) sphere-like tensegrity structures are used for the NSK, with cables (cyan elements) representing chromatin and with the nuclear lamina modelled as struts (purple elements). Both tensegrity structures describing the NSK are symmetrical since they are derived from the cuboctahedron and octahedron. The centre of the NSK coincides with the centre of the corresponding CSK, and each node of the NSK is connected to the node of the CSK pointing the same direction by IFs treated as the linker, as demonstrated in Figures 3(c) and 3(d).

### 2.2. Material Properties and Boundary Conditions

Although most components of the cell exhibit more or less nonlinear constitutive behaviour, all materials are assumed to be linear elastic for simplicity. Moreover, the material parameters of parylene C are used for the microplates because this material has the advantages of ease of manufacturing and biocompatibility and is commonly used in the microfabrication [35]. The material and geometrical properties for all the components are based on the values published in the literature and summarized in Table 1.

The cables and struts are depicted as truss elements that support only axial force and deformation, neglecting subcellular bending. The prestress carried in a cable (*F*) with a current length (*l*) is [10]

(2) $$\begin{array}{c} F=\left\{\begin{array}{cc} F_{0}+E_{a}A_{a}\left(\frac{l-l_{0}}{l_{r}}\right), & \text{if }l>l_{r},\\ 0, & \text{if }l\leq l_{r}, \end{array}\right. \end{array}$$

where *l*_*r*_ and *l*_0_ denote the resting and initial cable lengths, respectively, and *E*_*a*_ and *A*_*a*_ are Young's modulus and cross-section area of cables, respectively. *F*_0_ is the initial cable tension, described as follows:

(3) $$\begin{array}{c} F_{0}=\frac{E_{a}A_{a}}{l_{r}}\left(l_{0}-l_{r}\right). \end{array}$$

Meanwhile, the prestress carried in a strut (*P*) with a current length (*L*) is

(4) $$\begin{array}{c} P=\left\{\begin{array}{cc} P_{0}+E_{s}A_{s}\left(\frac{L_{r}-L}{L_{r}}\right), & \text{if }L<L_{r},P<P^{\text{c}},\\ 0, & \text{if }L\geq L_{r}, \end{array}\right. \end{array}$$

where *L*_*r*_ and *L*_0_ denote the resting and initial lengths of struts, respectively, and *E*_*s*_, *B*_*s*_, and *A*_*s*_ are Young's modulus, bending stiffness, and cross-section area of struts, respectively. *P*_0_ and *P*_*c*_ are the initial strut tension and axial thrust, respectively, described as

(5) $$\begin{array}{c} P_{0}=\frac{E_{s}A_{s}}{L_{r}}\left(L_{r}-L_{0}\right),\\ P^{c}=\frac{\pi^{2}B_{s}}{L_{r}^{2}}. \end{array}$$

The initial boundary conditions for the cell tensegrity structure models are that the 12-node tensegrity structure has three nodes pinned to the microplate and three cables located on the microplate and coupled with the microplate, while the 24-node tensegrity structure has four nodes and four cables. The nodes closest to the microplate are anchored to the corresponding nodes of the microplate via spring elements, and the other nodes are pinned as free moveable joints. The number of nodes on the microplate is determined according to the *z* coordinate of the node. If *z* is equal to 0, it is on the microplate, and if *z* is greater than 0, the node is not on the microplate. The smaller the *z* is, the closer it is to the microplate. The centre of the microplate is constrained in all degrees of freedom. A concentrated force of 10 pN is applied at the farthest nodes parallel to the microplate [40]. Only one truss element is used for all subcellular components, and the microplate is meshed with 8-node hexahedral elements.

## 3. Results

### 3.1. Influence of the Level of Complexity on the Folding Angle

**Figure 4:**
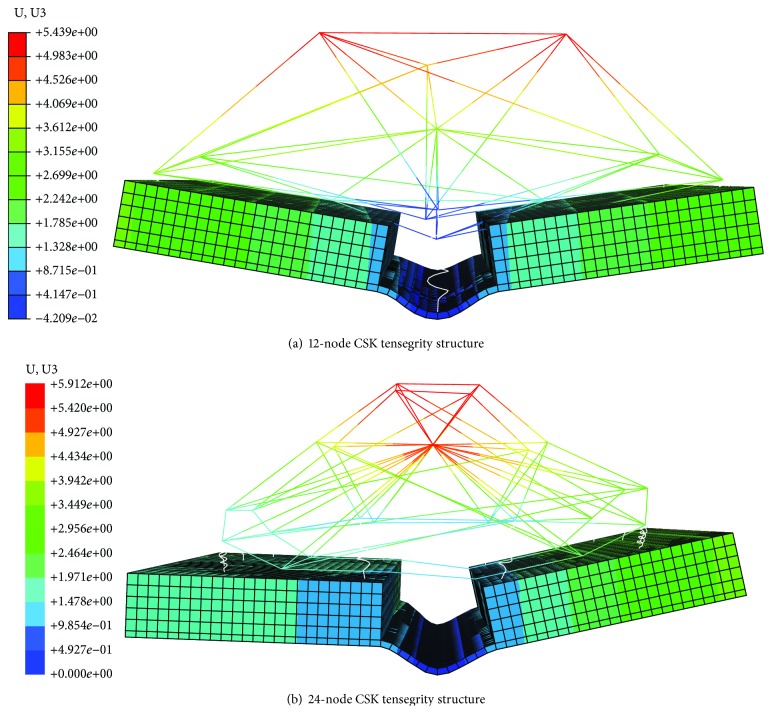
Schematic diagram of cell origami without the NSK.

Since Kaori et al. have shown that the folding angle of the microplate is related to the cell types [1], the relationship between the folding angle and levels of the complexity tensegrity structure in the cell origami process is studied using two different levels of complexity for the CSK and NSK. A deformation diagram of cell origami without the NSK is shown in Figure 4.

**Figure 5:**
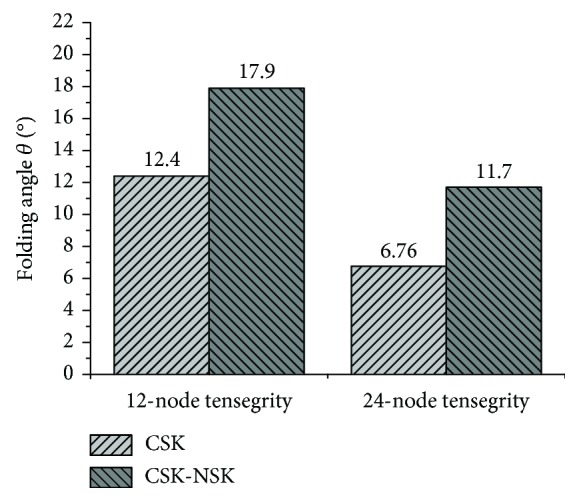
The maximum folding angle of the microplate.

The maximum folding angles of the microplates of the 12-node and the 24-node CSK models are 12.4° and 6.76°, respectively, and the maximum values of the 12-node and the 24-node CSK-NSK models are 17.9° and 11.7°, respectively, as demonstrated in Figure 5.

The result shows that the stiffness of the 12-node tensegrity structure model with/without the NSK is larger than that of the 24-node model with/without the NSK, indicating indirectly that the folding angle is related to the cell type from the perspective of simulation. This result may be due to the larger number of nodes, the greater complexity of the structure, the additional degree of freedom, the additional energy required for deformation, and the smaller folding angle of the microplate. Furthermore, the folding angle is asymmetrical, which may be caused by the asymmetry of the model, as shown in Figure 4.

### 3.2. Effect of the NSK on the Folding Angle

**Figure 6:**
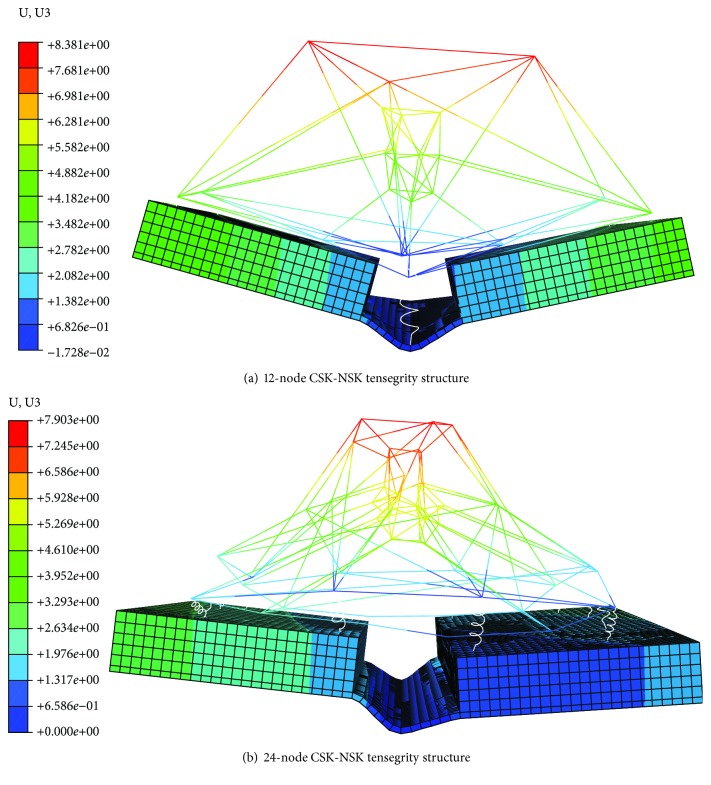
Schematic diagram of cell origami with the NSK.

In view of the fact that the nucleus represented by the NSK has a large volume occupancy and includes genetic information, to investigate the effect of the NSK structure on the folding angle, two tensegrity structure models with different levels of NSK complexity are established and compared with the model without the NSK. The deformation diagram of cell origami with the NSK is shown in Figure 6.

The maximum folding angle of the model with the NSK is larger than that without the NSK, as shown in Figure 5. The results show that the augmentation of the NSK can increase the stiffness of the model, independent of the complexity. An increase in stiffness means an increase of the folding angle, indicating that the cell is closer to its physiological environment. The above results imply that the 24-node tensegrity structure is sufficiently complex to describe the flat tensegrity structure representing the adherent cell morphology, and the results are consistent with the conclusions of Pugh [41], who demonstrated that when the levels of complexity of the structure increase further, the tensegrity structure becomes more analogous to a cylinder and does not represent the geometry of suspended cells.

## 4. Discussions

The proposed tensegrity structure models of adherent cells is aimed at better understanding the cell origami. In comparison with cells, the cables of the cellular tensegrity structure may be viewed as analogous to the CSK tension elements (e.g., MFs), the struts as the CSK compression elements (e.g. MTs), the microplate as the ECM, and spring elements as the FACs. Since the tensegrity structure is a simplification of the CSK morphology, the FEM models help us to understand the cell origami in a simple way, which provide an attempt to measure the cell traction force in a 3D physiological environment and a new method for further study on cell origami. Unfortunately, the simulation of cell origami has many limitations compared with the existing cell models and the folding of living cells. The main limitations can be succinctly summarized as follows.

First, some studies have shown that a more complex computational model, such as bendo-tensegrity models, can better understand the mechanotransduction mechanism and can be used to determine the mechanical contribution of individual cytoskeletal components to the cellular overall structural responses [42]. Therefore, although the tensegrity structure contains many features consistent with living cells and could be used to represent a reasonable starting point for describing CSK mechanics, it is an oversimplification of the CSK morphology. The following work is to establish a more sophisticated and accurate adherent cell model to describe the cell origami. Second, although some studies have shown that the mechanical properties of IFs are far from linear elastic and that IFs and FACs show obvious strain stiffening behaviour [33, 43], for simplification, the strain stiffening behaviours of IFs and FACs are not considered and the mechanical properties of IFs and FACs are still assumed to be linear elastic, which is consistent with the actual situation. Third, some studies have shown that MTs of unequal lengths originate from centrosomes near the nucleus and spread outward through the cytoplasm to the cell cortex where they interact with other cytoskeletal filaments at FACs [42], while others have shown that cytoplasmic IFs are radially distributed from the nuclear membrane towards the cell surface [34]. In view of the fact that the distribution of CSK varies with the cell type, the following work is to create a different CSK model to describe cell origami. Finally, although most cellular components behave more or less in a nonlinear constitutive behaviour, linear elastic properties are assigned to all components of the model, which is far from the real behaviour of cells.

Therefore, the present model can be further improved by considering the more complex and different distribution of FEM modelling of the CSK and NSK with viscoelasticity or hyperelasticity properties, so that we can better understand the mechanotransduction mechanism and accurately measure the traction force of living cells through the folding angle of the microplate.

## 5. Conclusions

In the present study, a 3D FEM model of adherent cells with different levels of tensegrity structure complexity is developed. The cell origami model is constructed on the basis of the principle of the origami folding technique and cell traction forces. The process of cell origami is first performed by using the spring elements to connect the model with the microplate in order to fold a microplate from a 2D configuration to form a 3D cell-populated microstructure. The simulation results are as follows:

- The inclusion of the NSK can enhance the folding angle of the microplate. The larger the folding angle is, the closer it is to the real situation of the cells in the 3D environment, which cannot be described in the 2D environment
- Increasing the level of complexity of the model can reduce the folding angle, indirectly demonstrating that the folding angle depends on the cell type from the perspective of simulation

In other words, both the level of complexity of the tensegrity structures and the NSK have an important influence on the behaviour of cell origami. The proposed FEM models can provide theoretical guidance for the application of biotechnology and the analysis of the 3D structures of cells and have a great potential to be implemented for the self-assembly of cell-based microscale medical devices.

## Figures and Tables

**Table 1:** The material parameters and geometric dimensions.

	Elastic modulus (Pa)	*ν*	Dimensions
MTs [36]	1.2 × 10^9^	0.3	190 nm^2^
MFs [36]	2.6 × 10^9^	0.3	19 nm^2^
IFs [37]	2 × 10^9^	0.3	100 nm^2^
Lamina [38]	1.4 × 10^6^	0.3	78.5 nm^2^
Chromatin [38]	244 × 10^6^	0.3	1.13 nm^2^
Microplate [1]	4 × 10^9^	0.3	30 × 30 × 2.7 *μ*m^3^
Flexible joint [1]	4 × 10^9^	0.3	30 × 6 × 0.3 *μ*m^3^
Bond stiffness [39]	*k*_*b*_ = 0.025 nN/*μ*m		

## Data Availability

All data used and analyzed during the current study are available from the corresponding author on reasonable request.

## References

[1] Kuribayashi-Shigetomi K., Onoe H., Takeuchi S. (2012). Cell origami: self-folding of three-dimensional cell-laden microstructures driven by cell traction force. *PLoS One*.

[2] Davis D., Chen B., Dickey M. D., Genzer J. (2016). Self-folding of thick polymer sheets using gradients of heat. *Journal of Mechanisms and Robotics*.

[3] Azam A., Laflin K. E., Jamal M., Fernandes R., Gracias D. H. (2011). Self-folding micropatterned polymeric containers. *Biomedical Microdevices*.

[4] Sirrine J. M., Pekkanen A. M., Nelson A. M., Chartrain N. A., Williams C. B., Long T. E. (2015). 3D-printable biodegradable polyester tissue scaffolds for cell adhesion. *Australian Journal of Chemistry*.

[5] Song J., Zhu G., Gao H. (2018). Origami meets electrospinning: a new strategy for 3D nanofiber scaffolds. *Bio-Design and Manufacturing*.

[6] He Q., Okajima T., Onoe H., Subagyo A., Sueoka K., Kuribayashi-Shigetomi K. (2018). Origami-based self-folding of co-cultured NIH/3T3 and HepG2 cells into 3D microstructures. *Scientific Reports*.

[7] Maskarinec S. A., Franck C., Tirrell D. A., Ravichandran G. (2009). Quantifying cellular traction forces in three dimensions. *Proceedings of the National Academy of Sciences of the United States of America*.

[8] Stamenović D., Ingber D. E. (2002). Models of cytoskeletal mechanics of adherent cells. *Biomechanics and Modeling in Mechanobiology*.

[9] Chen T.-J., Wu C.-C., Tang M.-J., Huang J.-S., Su F.-C. (2010). Complexity of the tensegrity structure for dynamic energy and force distribution of cytoskeleton during cell spreading. *PLoS One*.

[10] Coughlin M. F., Stamenovic D. (1998). A tensegrity model of the cytoskeleton in spread and round cells. *Journal of Biomechanical Engineering*.

[11] Ingber D. E., Dike L., Hansen L. (1994). Cellular tensegrity: exploring how mechanical changes in the cytoskeleton regulate cell growth, migration, and tissue pattern during morphogenesis. *International Review of Cytology*.

[12] Ingber D. E. (2008). Tensegrity-based mechanosensing from macro to micro. *Progress in Biophysics and Molecular Biology*.

[13] Ingber D. E. (2006). Cellular mechanotransduction: putting all the pieces together again. *Faseb Journal Official Publication of the Federation of American Societies for Experimental Biology*.

[14] Chen L., Jiang F., Qiao Y. (2018). Nucleoskeletal stiffness regulates stem cell migration and differentiation through lamin A/C. *Journal of Cellular Physiology*.

[15] Bursa J., Lebis R., Holata J. (2012). Tensegrity finite element models of mechanical tests of individual cells. *Technology and Health Care*.

[16] Makhija E., Jokhun D. S., Shivashankar G. V. (2016). Nuclear deformability and telomere dynamics are regulated by cell geometric constraints. *Proceeding of the National Academy of Sciences of the United States of America*.

[17] Mcgarry J. G., Prendergast P. J. (2004). A three-dimensional finite element model of an adherent eukaryotic cell. *European Cells and Materials*.

[18] Parameswaran H., Lutchen K. R., Suki B. (2014). A computational model of the response of adherent cells to stretch and changes in substrate stiffness. *Journal of Applied Physiology*.

[19] Stamenović D., Fredberg J. J., Wang N., Butler J. P., Ingber D. E. (1996). A microstructural approach to cytoskeletal mechanics based on tensegrity. *Journal of Theoretical Biology*.

[20] Coughlin M. F., Stamenović D. (1997). A tensegrity structure with buckling compression elements: application to cell mechanics. *Journal of Applied Mechanics*.

[21] Stamenović D., Coughlin M. F. (1999). The role of prestress and architecture of the cytoskeleton and deformability of cytoskeletal filaments in mechanics of adherent cells: a quantitative analysis. *Journal of Theoretical Biology*.

[22] Stamenovic D., Coughlin M. F. (2000). A quantitative model of cellular elasticity based on tensegrity. *Journal of Biomechanical Engineering*.

[23] Wendling S., Oddou C., Isabey D. (1999). Stiffening response of a cellular tensegrity model. *Journal of Theoretical Biology*.

[24] Wang N., Stamenović D. (2000). Contribution of intermediate filaments to cell stiffness, stiffening, and growth. *American Journal of Physiology-Cell Physiology*.

[25] Volokh K. Y., Vilnay O., Belsky M. (2000). Tensegrity architecture explains linear stiffening and predicts softening of living cells. *Journal of Biomechanics*.

[26] Abolfathi N., Karami G., Ziejewski M. (2008). Biomechanical cell modelling under impact loading. *International Journal of Modelling and Simulation*.

[27] Sultan C., Stamenović D., Ingber D. E. (2004). A computational tensegrity model predicts dynamic rheological behaviors in living cells. *Annals of Biomedical Engineering*.

[28] Bursa J., Lebis R., Janicek P. (2006). FE Models of stress-strain states in vascular smooth muscle cell. *Technology and Health Care*.

[29] Peng K. Y. (2016). *A Computational Model of Cell Movement on Surface with Concave Corner Architecture and Viscoelastic Effects*.

[30] Hartman C. D., Isenberg B. C., Chua S. G., Wong J. Y. (2017). Extracellular matrix type modulates cell migration on mechanical gradients. *Experimental Cell Research*.

[31] Wang N., Stamenović D. (2002). Mechanics of vimentin intermediate filaments. *Journal of Muscle Research and Cell Motility*.

[32] Janmey P. A., Euteneuer U., Traub P., Schliwa M. (1991). Viscoelastic properties of vimentin compared with other filamentous biopolymer networks. *Journal of Cell Biology*.

[33] Nolting J.-F., Möbius W., Köster S. (2014). Mechanics of individual keratin bundles in living cells. *Biophysical Journal*.

[34] Djaball K. (1999). Cytoskeletal proteins connecting intermediate filaments to cytoplasmic and nuclear periphery. *Histology and Histopathology*.

[35] de la Oliva N., Mueller M., Stieglitz T., Navarro X., del Valle J. (2018). On the use of parylene C polymer as substrate for peripheral nerve electrodes. *Scientific Reports*.

[36] Voloshin A. (2016). Modeling cell movement on a substrate with variable rigidity. *International Journal of Biomedical Engineering and Science*.

[37] Ghaffari H., Saidi M. S., Firoozabadi B. (2017). Biomechanical analysis of actin cytoskeleton function based on a spring network cell model. *Proceedings of the Institution of Mechanical Engineers, Part C: Journal of Mechanical Engineering Science*.

[38] Xue F., Lennon A. B., McKayed K. K., Campbell V. A., Prendergast P. J. (2015). Effect of membrane stiffness and cytoskeletal element density on mechanical stimuli within cells: an analysis of the consequences of ageing in cells. *Computer Methods in Biomechanics and Biomedical Engineering*.

[39] He S., Su Y., Ji B., Gao H. (2014). Some basic questions on mechanosensing in cell–substrate interaction. *Journal of the Mechanics and Physics of Solids*.

[40] van Loon J. J. W. A. (2009). Mechanomics and physicomics in gravisensing. *Microgravity Science and Technology*.

[41] Pugh A. (1976). *An Introduction to Tensegrity*.

[42] Bansod Y. D., Matsumoto T., Nagayama K., Bursa J. (2018). A finite element bendo-tensegrity model of eukaryotic cell. *Journal of Biomechanical Engineering*.

[43] Huang H., Kamm R. D., So P. T. C., Lee R. T. (2001). Receptor-based differences in human aortic smooth muscle cell membrane stiffness. *Hypertension*.

